# Successful resuscitation from acute coronary syndrome during mediastinoscope-assisted transhiatal esophagectomy: a case report

**DOI:** 10.1186/s44215-023-00086-7

**Published:** 2023-08-01

**Authors:** Kiyotomi Maruyama, Kou Shimada, Arano Makino, Ryo Hisamune, Masanori Kawaguchi, Shigeo Ikeno, Natsuhiro Morita, Ken Ichioka, Tadaaki Shimizu, Tomoki Shirota, Kuniyuki Gomi, Motohiro Mihara, Shoji Kajikawa

**Affiliations:** 1 Department of Surgery, Suwa Red Cross Hospital, 5-11-50 Kogandoori, Suwa, 392-0027 Japan; 2 Department of Cardiology, Suwa Red Cross Hospital, 5-11-50 Kogandoori, Suwa, 392-0027 Japan; 3 Department of Anesthesiology, Suwa Red Cross Hospital, 5-11-50 Kogandoori, Suwa, 392-0027 Japan

**Keywords:** Mediastinoscopic surgery, Esophageal cancer, Cardiac arrest

## Abstract

**Background:**

Although unexpected cardiac arrest is a very rare intraoperative complication, strategies regarding preoperative screening and procedures to be taken in the event of an emergency need to be well established.

**Case presentation:**

A man in his late 70 s diagnosed with thoracic esophageal cancer, cT3N1M0, and cStage III was admitted. His metabolic equivalents were 4 or more. Electrocardiogram (ECG), ultrasound cardiography, and hematological examinations revealed no severe abnormalities. Computed tomography (CT) showed highly calcified coronary arteries. We performed mediastinoscope-assisted transhiatal esophagectomy. Procedures in the mediastinum involving access from the neck and abdomen were completed uneventfully. Middle mediastinal lymph node dissection and gastrointestinal reconstruction with the patient in the prone position were scheduled for later. However, before the change in position, pulseless nonsustained ventricular tachycardia (VT) suddenly occurred and caused blood pressure to drop sharply to below 30 mmHg. The VT disappeared in approximately 20 s, and there was a return to sinus rhythm after cardiac resuscitation; however, the ECG showed a decrease in the ST segments of leads II, III, and aVF. Immediately thereafter, the patient was transferred to the cardiac catheterization laboratory for percutaneous coronary intervention. Cardiac catheterization revealed diffuse stenosis of 90% in the left anterior descending branch of the coronary artery (segment no. 6). Plain old balloon angioplasty and stent placement were performed. Dual antiplatelet therapy was needed. On the next day, thoracoscopic esophagectomy was performed in the left lateral decubitus position, followed by cervical esophagostomy in the supine position. He developed acute respiratory distress syndrome and thoracic aortic dissection on the 5th postoperative day (POD) and intraperitoneal bleeding on the 16th POD. On the 105th POD, laparoscopic-assisted cervical esophagogastric anastomosis was performed. Parkinson’s disease was diagnosed on the 126th POD. On the 313th POD, the patient was discharged.

**Conclusion:**

Surgeons should be familiar with the guidelines for patient screening and management of intraoperative cardiac arrest. In patients with severe coronary artery calcification, further investigation such as coronary angiography CT may be necessary before esophagectomy. Furthermore, highly invasive surgery should be performed in well-equipped hospitals.

## Background

The prevalence of thoracoscopic and laparoscopic surgery, understanding of anatomy, and advances in surgical techniques and anesthesia have made it possible to provide safer and less invasive surgical treatments for patients. However, with the aging of patients, cases with preoperative complications increase, and there is a certain probability of life-threatening intraoperative complications occurring. It has been reported that there are 1.05–2.73/million cases of intraoperative arrest [1–3] and 5.62–7.36/million cases of perioperative cardiac arrest [4, 5]. Although unexpected cardiac arrest is a very rare intraoperative complication, strategies regarding preoperative screening and measures in the event of an emergency need to be well established.

Mediastinoscope-assisted transhiatal esophagectomy (MATE) is performed in some institutions and is emphasized as the ultimate minimally invasive esophageal surgery, avoiding chest wall trauma, collapse of the lung, and reduction in pulmonary function [6, 7]. It is a surgical procedure that may become as widely used in the future as the thoracoscopic approach given progress in understanding mediastinal anatomy as viewed from the left neck. The perioperative outcomes of this procedure are not specific and are not inferior to those of the thoracoscopic approach [8].

In Japan, clinical societies have published guidelines for treating intraoperative cardiac arrest as well as guidelines for screening and management of preoperative cardiovascular complications. The former publication is the “Practical Guide for Intraoperative Cardiac Arrest” published by the Japanese Society of Anesthesiologists (JSA) [9], and the latter, published by the Japanese Circulation Society (JCS) and the Japanese College of Cardiology, is “The JCS 2022 Guideline on Perioperative Cardiovascular Assessment and Management for Noncardiac Surgery” [10]. In managing patients with cancer, practitioners should be familiar with these guidelines and institute perioperative management appropriate to the patient’s background; daily surgery should not be performed carelessly.

In this article, we report a patient with intraoperative cardiac arrest during MATE for esophageal cancer who was subsequently successfully treated with percutaneous coronary intervention (PCI) even though treating acute respiratory distress syndrome (ARDS) and Parkinson’s disease afterward was difficult. There has been no previous report about intraoperative cardiac arrest in esophagectomy that present information in detail.

## Case presentation

**Fig. 1:**
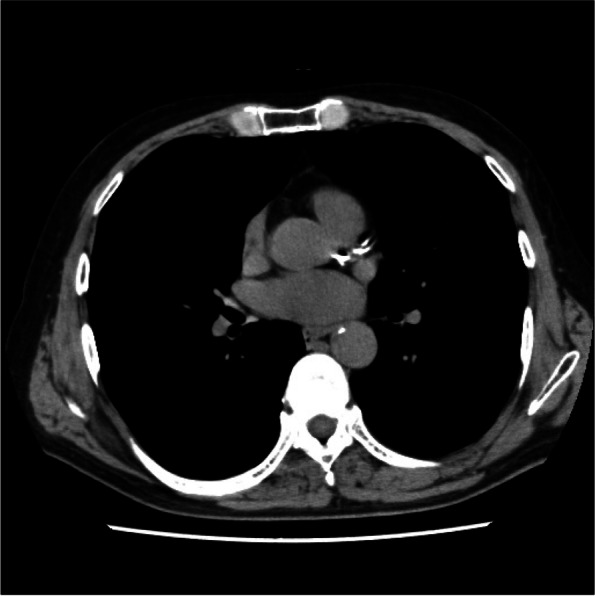
Computed tomography showed highly calcified coronary artery

A man in his late 70 s had experienced a feeling of stuffiness during swallowing for 2 months before his visit to his family doctor. Upper gastrointestinal endoscopy indicated a diagnosis of esophageal cancer, and the patient was referred to our hospital. He has history of drinking 180-mL distilled liquor daily but no smoking history. His past medical history included hyperuricemia from the age of 40, no diabetes, and no hypertension. He and his family had no history of cardiovascular disease. His performance status (PS) and American Society of Anesthesiologists physical status were 1 and 2, respectively. His metabolic equivalents (METs) were 4 or more. Complete blood count, biochemical examination, and urinalysis showed no abnormalities with the exception of a serum albumin level of 3.8 g/dL (Table 1). Spirogram examination showed forced vital capacity (FVC) of 3520 mL, forced expiratory volume in 1 s (*FEV*_1.0_) of 2090 mL, FEV_1.0_/FVC of 59.4%, and mild obstructive pulmonary dysfunction. Electrocardiogram (ECG) examination revealed a heart rate of 56 beats/min and no abnormalities with the exception of supraventricular extrasystoles. Ultrasound cardiography (UCG) revealed an ejection fraction of 73.2%, no abnormalities in cardiac wall motion, and no cardiac valve abnormalities. Computed tomography (CT) showed highly calcified coronary arteries (Fig. 1).

**Table 1:** Complete blood count and biochemical examination

WBC	5290/μL	ChE	211 U/L	PT	12.0 s (102%)
RBC	391 × 10^4^/μL	LAP	46 U/L	INR	0.99
Hb	13.9 g/dL	CK	138 U/L	APTT	34.3 s
Hct	38.5%	AMY	81 U/L	FDP	2.5 μg/mL
Plt	9.8 × 10^4^/μL	T-Cho	203 mg/dL	D-dimer	0.7 μg/mL
		TG	97 mg/dL		
TP	7.2 g/dL	UA	5.4 mg/dL	CEA	3.6 ng/mL
Alb	3.8 g/dL	UN	9.5 mg/dL	SCC	1.5 ng/mL
AST	20 U/L	Cre	0.77 m/dL	SYFRA	< 1.0 ng/mL
ALT	17 U/L	Na	141 mmol/L		
LDH	214 U/L	K	3.8 mmol/L		
ALP	216 U/L	Cl	105 mmol/L		
γ-GTP	14 U/L	BS	105 m/dL		
T-Bil	0.78 m/dL	HbA1c	5.5%

Examinations including esophagogram, esophageal endoscopy, and CT indicated a diagnosis of middle and lower thoracic esophageal cancer, type 4, 35 mm, cT3N1M0, cStage III (TNM Classification of Malignant Tumors, Eighth Edition) (TNM), and moderately differentiated squamous cell carcinoma. Since the patient was over 75 years old, surgical therapy was performed without neoadjuvant chemotherapy. We planned MATE as below. Lymphadenectomy in the upper mediastinum was performed under mediastinoscopy from the left neck, and laparoscopic lymphadenectomy was performed in the lower mediastinum and abdomen, followed by gastric tube preparation in the supine position. The plan was to progress to having the patient in a prone position for thoracoscopic lymphadenectomy in the middle mediastinum and esophagectomy, followed by intrathoracic esophagogastrostomy. Methylprednisolone was to be injected at a rate of 250 mg/day from day 0 to day 2 of the perioperative period to prevent systemic inflammatory response syndrome.

**Fig. 2:**
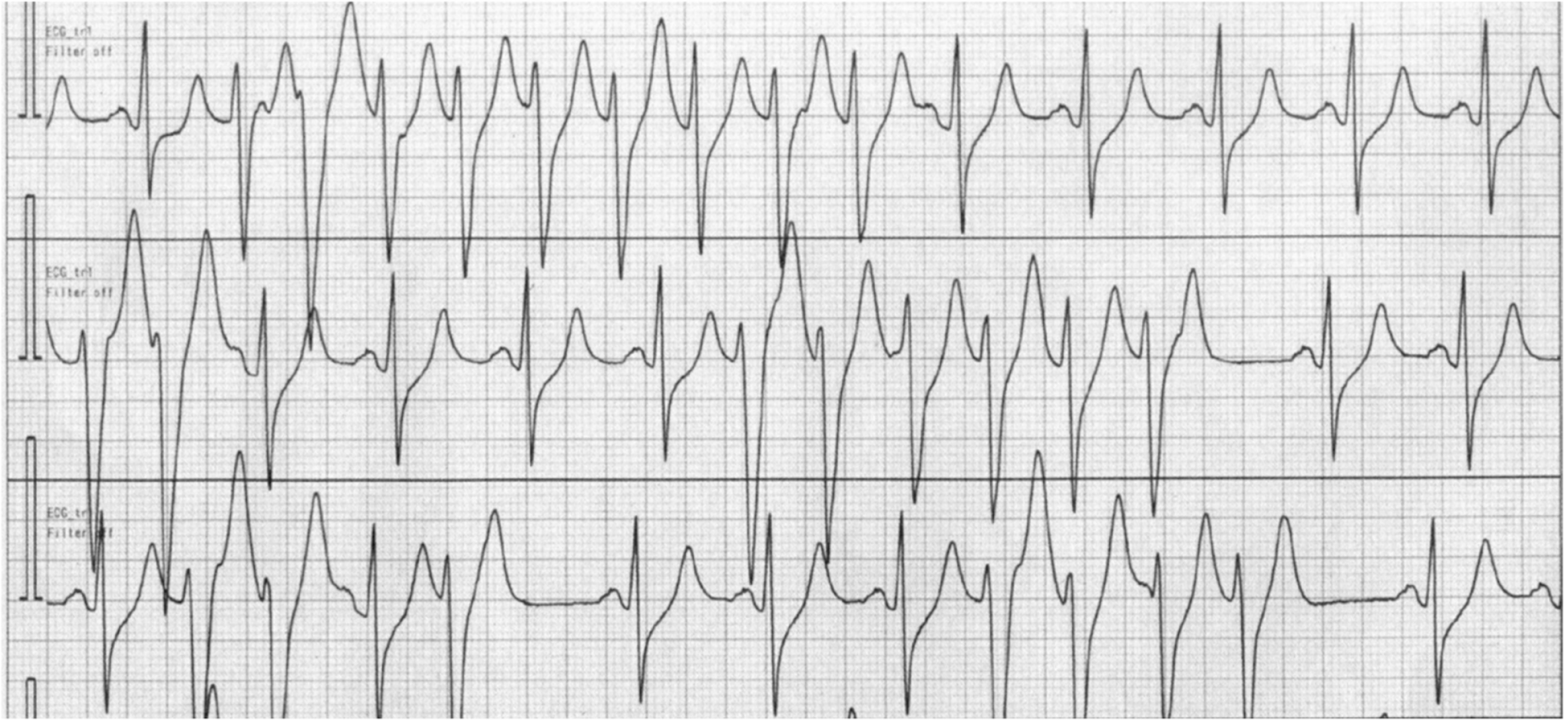
Electrocardiogram monitor (II lead) in the operation room showed ventricular tachycardia

**Fig. 3:**
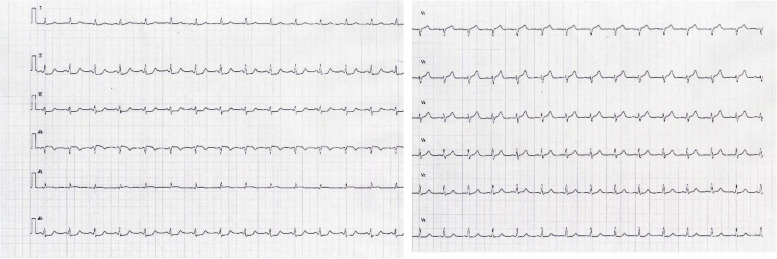
Electrocardiogram after the ventricular tachycardia disappeared showed a decrease in ST segment in the II, III, aVF leads

Procedures in the mediastinum involving access from the neck and abdomen were completed uneventfully as planned. Almost all the esophagus, with the exception of the middle portion, was dissected, and the esophagus was severed at the esophagogastric junction. The operation time was 250 min, and blood loss was 420 g. However, pulseless nonsustained ventricular tachycardia (NSVT) suddenly occurred and caused the patient’s blood pressure to drop sharply to below 30 mmHg before the change to a prone position (Fig. 2). Immediately, we called in a cardiologist while performing chest compressions. At the same time, lidocaine, dopamine hydrochloride, and nicorandil were intravenously administered. The VT disappeared in approximately 20 s, and there was a return to sinus rhythm, but the subsequent ECG showed a decrease in the ST segments of leads II, III, and aVF (Fig. 3). Immediately thereafter, the patient was moved to the cardiac catheterization laboratory for PCI under total anesthesia. The infusion volume until cardiac arrest was 1400 mL/6 h (3.8 mL/kg/h).

**Fig. 4:**
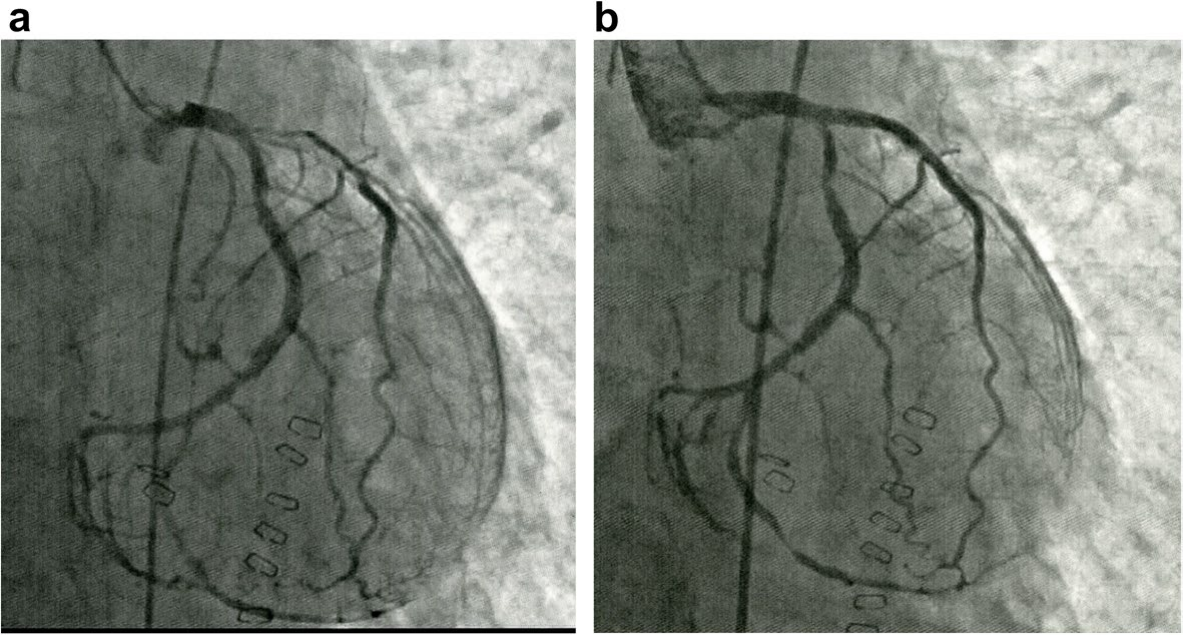
Coronary angiography before the PCI revealed diffuse stenosis 90% of segment no. 6 in the left anterior descending branch. Coronary angiography after the PCI showed the dilated coronary artery

Coronary angiography revealed diffuse stenosis 90% of segment no. 6 in the left anterior descending branch of the coronary artery. First, plain old balloon angioplasty was performed at the relevant site, but since intravascular dissociation was induced, stent placement was performed (Fig. 4a, b). Dual antiplatelet therapy (DAPT) was needed. The PCI time was 92 min. After completing PCI, the patient was returned to the operating room and underwent gastrostomy. The operation time was 90 min, and blood loss was 0 g. Acetylsalicylic acid and clopidogrel treatments were started through the gastrostomy tube. In addition, heparin was administered intravenously.

Although it was difficult to evaluate cardiac function by UCG due to the mediastinal operation, it was judged that myocardial damage was small because there was no significant increase in creatinine kinase after PCI (Table 2). In addition, since the hemodynamics were stable with normal conditions, after discussion with the cardiologist, it was decided that surgery would be performed as long as it could be completed a short time. Intra-aortic balloon pumping was prepared for stent occlusion. On the day after the first operation, thoracoscopic esophagectomy was performed in the left lateral decubitus position without lymphadenectomy, followed by cervical esophagostomy in the supine position. Although pale bloody pleural effusion accumulated in the thoracic cavity, the intrathoracic procedure was completed without causing any active bleeding. The mediastinal pleura was incised cranially and caudally, the esophagus was transected at the cranial side of the azygos vein arch, and the esophagus was removed. In an effort to prevent bleeding, the remaining subcarinal and main bronchus lymph nodes were not removed. The operation time was 96 min, and blood loss was 880 mL. Combined with the surgery the day before, the lymph node dissection sites were the cervical paraesophageal, upper, middle and lower thoracic paraesophageal, right and left recurrent nerve, supradiaphragmatic, thoracic paraaortic, lesser curvature, infradiaphragmatic, in the esophageal hiatus of the diaphragm, along the left gastric artery, and along the celiac artery. The histopathological examination revealed squamous cell carcinoma, pT2N0M0, and pStage IIA (TNM).

**Table 2:** Laboratory date after PCI

	**2 h after**	**4 h after**	**8 h after**	**12 h after**	**16 h after**
WBC 10^2^/μL	80.1			104.8	
Hgb g/dL	6.4			11.4	
Plt 10^4^/μL	4.3			7.9	
AST U/L	35	40	48	50	60
ALT U/L	31			33	
CK U/L	171	213	292	596	712
CK-MB U/L	11	10	16	36	38
D-dimer μg/mL	41.25			11.94	
APTT s	128.2			45.2	
PT s	18.8			13.6	
IN INR	1.61			1.13	

**Fig. 5:**
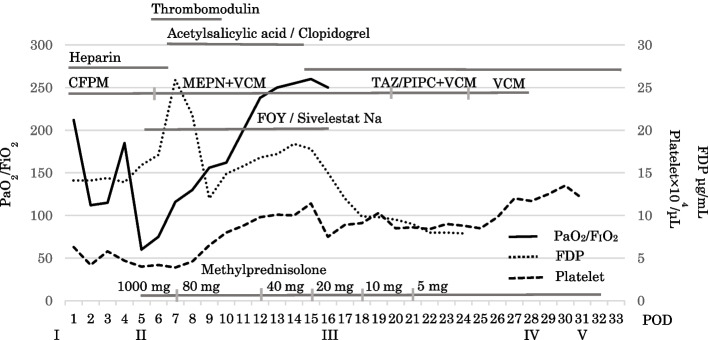
Postoperative clinical course. I, operation; II, aortic aneurysm; III, intraabdominal hemorrhage; IV, ventilator off; V, left the ICU; POD, postoperative day; CFPM, cefepime; MEPN, meropenem; VCM, vancomycin; TAZ/PIPC, tazobactam/piperacillin; FOY, gabexate mesilate

**Fig. 6:**
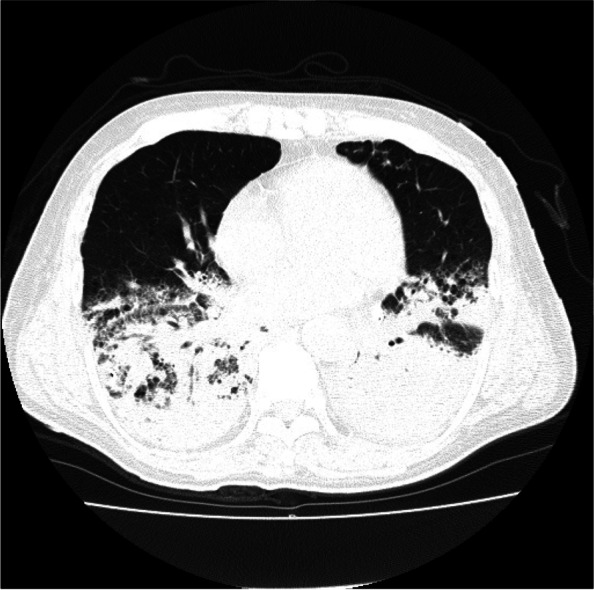
CT on the 5th POD revealed diffuse infiltration shadows in the bilateral lower lobe of the lung

**Fig. 7:**
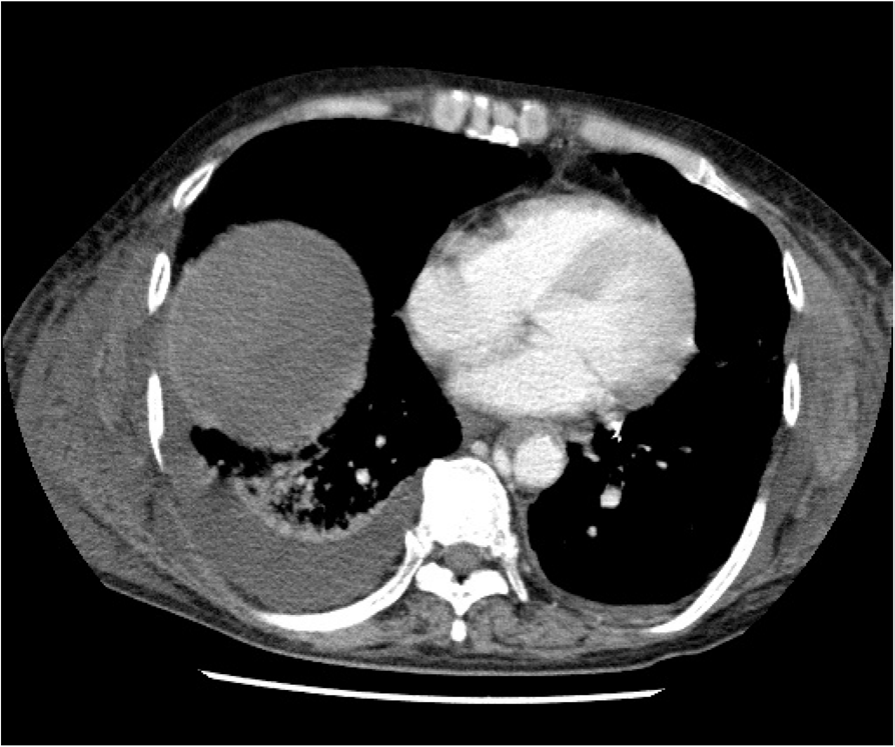
CT on the 5th POD revealed a dissection of the thoracic aortic aneurysm

**Fig. 8:**
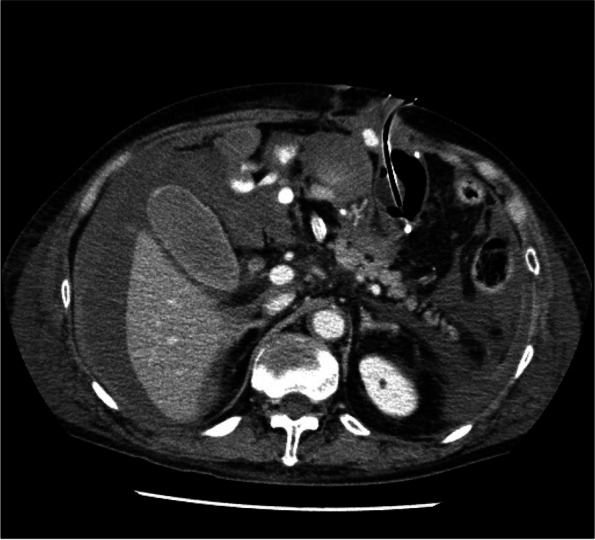
CT on the 16th POD revealed a large amount of bloody ascites due to active bleeding from the tube jejunostomy site

Postoperative management occurred in the intensive care unit (ICU) and involved use of a ventilator. Figure 5 shows the postoperative clinical course. Heparin and dual antiplatelet therapy were started on the 1st postoperative day (POD) and on the 6th POD. On the 5th POD, CT was performed because blood gas analysis showed deterioration of PaO2/FiO2 (*PaO2/FiO2* = 75), and chest X-rays showed the progression of infiltrative shadows in both lower lungs. CT revealed diffuse infiltration shadows in the bilateral lower lobe of the lung. The patient was diagnosed with severe ARDS (Fig. 6). Sputum culture revealed methicillin-resistant *Staphylococcus aureus*. At the same time, disseminated intravascular coagulation (DIC) developed (platelet count 4.2 × 10^4^/μL, FDP 25.9 μg/mL). In addition, CT performed for ARDS scrutiny revealed a dissection of the thoracic aortic aneurysm (Fig. 7). High-dose methylprednisolone, gabexate mesilate and sivelestat sodium, and thrombomodulin were administered to treat ARDS and DIC, and those conditions were controlled by the 12th POD. Aortic dissection was treated conservatively with strict control of blood pressure, and no deterioration was observed on subsequent CT. Furthermore, on the 16th POD, a rapid progression of anemia (Hb 8.6 g/dL) was observed, and CT revealed a large amount of bloody ascites due to active bleeding from the tube jejunostomy site (Fig. 8). Suture hemostasis was performed by emergency surgery on the same day. DAPT, which had been administered until then, was discontinued, and intravenous heparin administration was begun instead. The patient was weaned off the ventilator on the 28th POD, and he managed to leave the ICU on the 31st POD.

Resting tremor was more pronounced than when the patient entered the ICU, and there was no improvement after he left the ICU. Therefore, a neurologist was consulted on the 126th postoperative day, and the patient was diagnosed with Parkinson’s disease (PD). The patient started taking L-DOPA. After that, a great impact was seen on swallowing function, walking function, and activities of daily living.

On the 105th POD, laparoscopic-assisted cervical esophagogastrostomy was performed although via the antethoracic route. The operation time was 215 min, and blood loss was 350 g. On the 313th POD, the patient was transferred to a rehabilitation hospital. Irradiation to the mediastinum was planned but abandoned due to the occurrence of repeated severe complications and PS 3. The patient had dysphagia caused by vocal cord paralysis, gait disturbance due to sarcopenia and severe Parkinson’s disease, and repeated aspiration pneumonia; he was also unable to take food or medications orally. He died of pneumonia on the 795th day after the first operation *without cancer recurrence*.

## Discussion

Due to the aging of the population of prospective surgical patients and advances in medical technology, the number of operations in serious cases is increasing compared to before. According to the survey of incident cases by JSA, average values of incidence (per 10,000 cases) of intraoperative cardiac arrest in Japan were 4.38 (2004–2008), 3.27 (2009–2011), and 2.6 (2012–2018), and the rate is gradually decreasing [11, 12]. The 30-day mortality rate after intraoperative cardiac arrest was high, ranging from 58.7 to 63.1% [11]. In other countries, it has been reported that there were 1.05–5.7/million cases of intraoperative arrest [1–4] and 5.62–7.36/million cases of perioperative cardiac arrest [4, 5]. The mortality rate after cardiac arrest is 35.7–70.9% [1–5, 13–16]. Mortality due to anesthesia-related cardiac arrest has been reported to be 0.01–1.12/million [5, 16, 17].

There are various causes of cardiac arrest, and there have been many reports of associated massive bleeding, airway problems, anaphylaxis, drug overdose, and so on [15, 16]. Of the many causes, intraoperative acute coronary syndrome is the most serious and requires urgent action. Although it is an unexpected and rare complication, acute coronary syndrome should be suspected at onset and diagnosed immediately; absolutely, appropriate measures are needed. During general anesthesia, there are special conditions that differ from normal circumstances, and no patient compliance can be expected. Initial treatment must be started for an abnormality suspected because of circulatory monitor findings. JSA has published guidelines regarding intraoperative cardiac arrest resulting from various pathological conditions [9]. The guidelines explain the individual pathological conditions that cause cardiac arrest and provide concrete details on how to address them. Correspondence algorithms that can be used immediately in the operation room are listed for each pathological condition. In our case, it was considered that the cause of cardiac arrest was the decrease in blood flow in the stenosed coronary artery due to the decrease in circulating blood volume. *Infusion volume* up to the time of cardiac arrest was *3.8 mL/kg/h*. Compared to the general infusion volume in endoscopic esophageal surgery, the amount was extremely small, and it was considered to be the cause of the decreased blood flow in the stenosed coronary arteries.

Very few papers have presented evaluations of cardiac function in the prone position. There is a report that evaluated cardiac function in the prone position using single-photon emission computed tomography [18]. Accordingly, prone positioning induced significant changes in systolic and diastolic function as well as dyssynchrony. The negative effects of prone positioning were more severe in patients with poor baseline cardiac function. Furthermore, surgery for esophageal cancer involves lymph node dissection in the mediastinum, so it may be better to avoid surgery in the prone position in patients with a history of ischemic heart disease or with reduced cardiac function. The adverse effects of mediastinoscopy in the supine position on cardiac function are unknown. However, the laparoscopic procedures involving the mediastinum above the level of the inferior pulmonary veins require cardiac displacement, reduce venous return, and alter hemodynamics; these are common consequences. Cardiac compression during mediastinal procedures should be avoided whenever possible regardless of body position.

There is a report on how to differentially diagnose causes of cardiac arrest [12]. Cardiac arrest is relatively easy to recognize if an arterial pressure line is available, but if it is not available, cardiac arrest may not be obvious. End-tidal carbon dioxide partial pressure (ETCO_2_) is the most reliable predictor of cardiac arrest because it declines rapidly with decreasing cardiac output and specifically reflects circulatory collapse. A report analyzing systolic blood pressure, mean arterial pressure, heart rate, and ETCO_2_ for 40 min before cardiac arrest caused by trauma or sepsis found that ETCO_2_ gradually decreased 40 min before cardiac arrest and sharply decreased 10 min before cardiac arrest. In contrast, other parameters remained normal up to 2–5 min before cardiac arrest. In particular, cardiac arrest should be determined without hesitation at 15 mmHg or less [19]. Transthoracic or transesophageal echocardiography (TTE or TEE) by a specialist is convenient and useful in determining the cause of cardiac arrest as long as it does not interfere with resuscitation [20]. In our case, NSVT suddenly occurred and caused blood pressure to drop sharply to below 30 mmHg; approximately, 20 s after the start of chest compressions, the heartbeat returned to normal rhythm, and hemodynamics also normalized. A cardiologist performed TTE without delay. TTE showed no abnormalities in cardiac wall motion or cardiac output. However, an ECG revealed ST segment depression, so we diagnosed cardiogenic cardiac arrest. Unexpected bleeding and abnormalities in the respiratory system could be ruled out. Due to the presence of emphysema in the mediastinum as a result of the operation, a detailed examination by TTE was difficult. TEE was not possible because the esophagus was dissected. Fortunately, the cardiac catheterization room was vacant between scheduled examinations, so we were able to start PCI immediately.

Cardiac arrest in this case fortunately occurred in the supine position before the patient was placed in the prone position. Even when surgery is performed in the prone position, we generally opt for a posture that allows a quick transition from the prone position to the supine position in case of an emergency such as bleeding; that is, instead of having the patient fully supine with both arms raised cranially, the right arm is raised cranially, and the left arm is placed under the anterior chest. In an emergency, the left forearm can be pulled out to the patient’s right side so that the patient can be quickly shifted to the left lateral or supine position for thoracotomy or cardiac massage.

According to the JCS 2022 Guideline [10], approximately 10% of surgical procedures result in perioperative complications, of which approximately 42% are reported to be cardiovascular complications such as myocardial infarction and cerebral infarction. Approximately, half of these cardiovascular complications are considered preventable. The guideline newly clarifies the “algorithm of preoperative examination.” Starting with the “urgency of noncardiac surgery” (step 1), the “presence or absence of cardiovascular emergency” (step 2), “risk assessment of noncardiac surgery itself” (step 3), “risk assessment of perioperative event” (step 4), and “evaluation of exercise tolerance or measurement of BNP value, NT-pro BNP value” (step 5), emphasis is placed on risk stratification and planning of risk mitigation strategies for preoperative, intraoperative, and postoperative phases. It also clearly states how to select preoperative examinations that are neither excessive nor deficient. The revised cardiac risk index (RCRI) is often used for perioperative event risk assessment [21]. In our institution, we request cardiologists to evaluate cardiac function for patients with ischemic change in ECG, abnormality in UCG, a score of less than 4 METs, or 2 or more in the RCRI. We consult cardiologists for elderly patients, especially those over the age of 80, even if they do not meet the criteria. At the discretion of the cardiologist, additional tolerance ECG, coronary angiography, or more are added without delay.

Although our patient did not meet the criteria for referral to a cardiologist, from a retrospective perspective, CT angiography of the coronary arteries should have been performed because of the severe calcification of the coronary arteries. Our patient had no cardiovascular history and an RCRI of 1. In patients with *RCRI* = 1, the inhospital or postoperative 30-day cardiovascular event rate is 2.9%. A preoperative CT of our patient showed extensive calcification of the coronary arteries (CCA). Although there have been reports showing a relationship between CCA and cardiac complications, the positive predictive value is low. The absence of CCA was a reliable predictor of a favorable postoperative cardiac course [22]. In addition, many elderly patients commonly have CCA, and there is no rationale from a cost–benefit point of view to add cardiovascular examinations to all such patients. However, in patients scheduled for highly invasive surgery, patients with severe CCA may require further investigation such as coronary angiography CT before surgery.

Brute force preoperative cardiac assessment is inefficient and meaningless for extremely rare cases of intraoperative cardiac arrest. Rather, it is essential to formulate a countermeasure for when intraoperative cardiac arrest occurs. In particular, diseases that require highly invasive surgery, such as esophageal cancer, should be more commonly addressed in hospitals with well-equipped facilities corresponding to areas such as anesthesiology, cardiology, gastroenterology, plastic surgery, and radiology. Aggregation of cancer cases is essential for highly specialized medical care and risk management.

PD is a risk factor for postoperative complications and prolonged hospital stays. It is important that medical experts share awareness about perioperative problems and their management in patients with PD [23]. Our patient had no history of PD preoperatively. It was conceivable that highly invasive surgery and subsequent severe complications, especially infections, ventilator management under long-term sedation, administration of multiple drugs including steroids, and the resulting loss of muscle volume, accelerated the deterioration of PD. For the perioperative management of PD, continuous administration of PD drugs and intervention by a specialist is important to prevent deterioration. In our patient, the emergence of finger tremor during long-term sedation delayed the definitive diagnosis and delayed the initiation of treatment by a specialist. Levodopa is absorbed mainly in the proximal jejunum. Fortunately, it was possible to administer medication through the jejunostomy tube. PD are more prone to immobility and developing dysphagia, respiratory dysfunction, urinary retention, and psychiatric symptoms [24]. Early rehabilitation with early postoperative mobilization is strongly recommended, assessing swallowing ability frequently, encouraging incentive spirometry, performing bladder scans, avoiding urethral catheters, and providing aggressive physical therapy. In our patient, the initiation of rehabilitation was delayed. In particular, it was thought that the significant decrease in muscle volume and strength adversely affected the recovery from PD. In addition to exacerbation of PD, recurrent laryngeal nerve palsy and tracheostomy were likely to have contributed to his decreased swallowing function. His significant deterioration of swallowing function caused recurrent aspiration pneumonia and progressively less active. In addition, although we set the calorie intake from 1600 to 1800 kcal with nutritional support from the jejunostomy tube, no improvement in nutrition was obtained even after discharge. Adverse effects of interactions between PD drugs and drugs used in the perioperative period have also been reported [25]. In our patient, it was possible that drugs used to treat complications adversely affected PD drugs.

## Conclusion

Japanese surgeons should be familiar with the guidelines for patient screening and intraoperative cardiac arrest. In patients with severe CCA, further investigation such as coronary angiography CT may be necessary before esophagectomy. Furthermore, highly invasive surgery should be performed in well-equipped hospitals, and future hospital consolidation in cancer treatment is desirable.

## Data Availability

The datasets supporting the conclusions of this article are included within the article. A part of the datasets is available from the corresponding author on reasonable request.

## Abbreviations

ECG: Electrocardiogram
VT: Ventricular tachycardia
PCI: Percutaneous coronary intervention
POD: Postoperative day
MATE: Mediastinal-assisted transhiatal esophagectomy
JSA: The Japanese Society of Anesthesiologists
JCS: The Japanese Circulation Society
ARDS: Acute respiratory distress syndrome
PS: Performance status
METs: Metabolic equivalents
UCG: Ultrasound cardiography
CT: Computed tomography
TNM: TNM Classification of Malignant Tumors, Eighth Edition
NSVT: Non-sustained ventricular tachycardia
DAPT: Dual antiplatelet therapy
ICU: Intensive care unit
DIC: Disseminated intravascular coagulation
ETCO_2_: End-tidal carbon dioxide partial pressure
TTE: Transthoracic echocardiography
TEE: Transesophageal echocardiography
RCRI: Revised cardiac risk index
CCA: Calcification of the coronary arteries
PD: Parkinson’s disease
